# Quetiapine augmentation of prolonged exposure therapy in veterans with PTSD and a history of mild traumatic brain injury: design and methodology of a pilot study

**DOI:** 10.1186/s40779-020-00278-0

**Published:** 2020-10-08

**Authors:** Muhammad R. Baig, Robert D. Beck, Jennifer L. Wilson, Jennifer A. Lemmer, Adeel Meraj, Eric C. Meyer, Jim Mintz, Alan L. Peterson, John D. Roache

**Affiliations:** 1 Mental Health, South Texas Veterans Healthcare System, 116 A, 7400 Merton Minter Blvd, San Antonio, TX 78229 USA; 2 Polytrauma Rehabilitation Center, South Texas Veterans Healthcare System, San Antonio, TX USA; 3 Department of Psychiatry and Behavioral Sciences, University of Texas Health Science Center at San Antonio, San Antonio, TX USA; 4 Department of Veterans Affairs VISN 17 Center of Excellence for Research on Returning War Veterans, Waco, TX USA; 5 Central Texas Veterans Healthcare System, Waco, TX USA; 6 Department of Psychology and Neuroscience, Baylor University, Waco, TX USA; 7 Department of Epidemiology and Biostatistics, University of Texas Health Science Center at San Antonio, San Antonio, TX USA; 8 Department of Psychology, University of Texas at San Antonio, San Antonio, USA; 9 Department of Pharmacology, University of Texas Health Science Center at San Antonio, San Antonio, TX USA

**Keywords:** Quetiapine, Trauma-focused psychotherapy, Posttraumatic stress disorder, Mild traumatic brain injury, Veterans

## Abstract

**Background:**

Selective serotonergic reuptake inhibitors (SSRIs) are first-line pharmacologic treatments for patients with posttraumatic stress disorder (PTSD), but must be given over extended period of time before the onset of action. The use of SSRIs in PTSD patients with mild traumatic brain injury (mTBI) is problematic since SSRIs could exacerbate post-concussion syndrome (PCS) symptoms. VA/DOD guidelines identify trauma-focused psychotherapy as the best evidence-based treatment for PTSD, but overall effectiveness is limited by reduced levels of patient engagement and retention. A previous study from this research group suggested that quetiapine monotherapy, but not risperidone or valproate, could increase engagement in trauma-focused psychotherapy.

**Methods:**

We report the study protocol of a pilot study funded under the South-Central Mental Illness Research, Education, and Clinical Center pilot study program from the U.S. Department of Veterans Affairs. This randomized, open-label study was designed to evaluate the feasibility of completing a randomized trial of quetiapine vs. treatment as usual to promote patient engagement in PTSD patients with a history of mTBI.

**Discussion:**

We expect that the success of this ongoing study should provide us with the preliminary data necessary to design a full-scale randomized trial. Positive efficacy results in a full- scale trial should inform new VA guidelines for clinical practice by showing that quetiapine-related improvements in patient engagement and retention may be the most effective approach to assure that VA resources achieve the best possible outcome for veterans.

**Trial registration:**

NCT04280965.

## Background

Over 60% of the veterans who sustain traumatic brain injury (TBI) develop posttraumatic stress disorder (PTSD) [1–3]. Combat veterans with even mild TBIs are prone to persistent post-concussion syndrome (PCS) and significant social and occupational dysfunction [4–13]. There is considerable overlap of symptoms between PCS and PTSD [13].

With very strong evidence support, prolonged exposure (PE) is part of the standard care for PTSD patients in VA and DoD practice settings [13–18]. The major concern with PE treatment of PTSD is the potential to evoke emotions. As a result, there has been much hesitance to engage in PE therapy in the first place and to continue treatment for sufficient period of time [19–23]. Therefore, there is a need to improve patient engagement and retention [24–26].

The selective serotonin reuptake inhibitors (SSRIs) sertraline and paroxetine are the only two medications approved by the U.S. Food and Drug Administration for the treatment of PTSD [27–35]. However, an Institute of Medicine review concluded that SSRI efficacy is limited, particularly for men suffering from PTSD due to military combat exposure [36, 37]. Limited efficacy of sertraline and paroxetine for PTSD among veterans with comorbid mild TBI (mTBI) has led to a treatment as usual (TAU) practice of using multiple medications. Such a practice often fails to achieve symptom remission. More importantly, undesired side effects from polypharmacy (e.g., headache, dizziness, light headedness, daytime sedation and fatigue) are often indistinguishable from PCS symptoms, and may further complicate mTBI management [38–41].

Adjunctive use of risperidone [42] and valproate monotherapy [43] have been studied as pharmacologic treatment for PTSD, but none has been shown to be superior to placebo in relieving symptoms. In a small, placebo-controlled trial in PTSD veterans, monotherapy with the atypical antipsychotic agent quetiapine did report significantly reduce arousal and reexperiencing [44].

Recent considerations for the design of clinical trials focused on PTSD treatment have suggested that we identify target systems involved in fear extinction and explore medications that enhance the targeted approach of trauma-focused therapies [45–53]. Emotional processing theory postulates that fear activation is an essential component of successful PTSD treatment, and the extent of emotional reactions during trauma-focused therapy (TFT) has been associated with the magnitude of clinical improvement [54]. However, it is not clear whether physiologic arousal that occurs with emotional reactions per se is a necessary component of fear extinction or merely a secondary consequence of the more critical component of extinction involving fear-related stimuli under the safe conditions of therapy [55]. Previous studies suggested that psychosedation with benzodiazepines may be counter-therapeutic because of impaired fear extinction learning during virtual reality-based exposure therapy [56–58].. However, these negative effects of benzodiazepines could be due to γ-aminobutyric acid (GABA)-mediated impairment of the learning that must occur with trauma-focused therapies [58].

Quetiapine is an atypical antipsychotic with a broad spectrum of actions at several receptors, including dopaminergic (D1, D2, D3 and D4), serotonergic (5-HT2A, 5-HT2C and 5-HT7), adrenergic (α1), histaminic (H1) and muscarinic (mACh) and partial agonist (functional antagonist) actions at 5-HT1A receptors [59]. It is approved for the treatment of schizophrenia [60], bipolar disorder [61] and as an adjunct to treat major depressive disorder [62]. A previous trial of quetiapine monotherapy in veterans with PTSD showed reduced arousal and reexperiencing symptoms though few patients achieved remission and improved avoidance [44]. Quetiapine attenuates irritability, anxiety and sleep disturbances without impairing sleep architecture, [63] and theoretically may benefit patients undergoing fear-extinction learning in trauma- focused psychotherapy [64]. We speculate that the anxiolytic and sleep-promoting effects of quetiapine [65] could attenuate the unpleasant anxiety and irritability associated with PE therapy without impairing fear extinction learning [63, 64, 66–69]. In the proposed study, we plan to examine whether quetiapine could enhance patient engagement in trauma-focused therapy.

In a previous observational study, we showed that quetiapine monotherapy, but not risperidone or valproate, increased engagement in therapy in 18 of 21 veterans with PTSD, and 50% of the patients with increased engagement achieved complete remission [70]. Unfortunately, local VA treatment guidelines specify that off-label use of atypical antipsychotics is permitted only after at least two failed trials of SSRIs. In the current report, we describe the study protocol of an ongoing, randomized, open-label trial that examined the feasibility of quetiapine vs. TAU on engagement in PE therapy in PTSD veterans with a history of mTBI.

There are three main hypotheses that pertain to changes in outcomes of interest during the treatment phase (weeks 1–12). Hypothesis 1 proposes that quetiapine could enhance engagement and retention in PE therapy, as measured by the number of sessions attended and self-report. Hypothesis 2 proposes that quetiapine could reduce PTSD severity at week 12, as measured by the total score on the PTSD Checklist for DSM-5 (PCL-5). Hypothesis 3 proposed that quetiapine could improve functional outcomes, as measured by the Quality of Life Scale (QOLS) and World Health Organization Disability Assessment Schedule 2.0 (WHODAS 2.0).

## Methods

### Ethics statement

The study protocol was approved and monitored by the University of Texas Health Science Center at San Antonio Institutional Review Board and STVHCS Research and Development Committee (HSC20180200H). The study was conducted in accordance with declaration of Helsinki. All study participants signed written informed consent and were eligible to receive other standard of care treatments.

### Study design and grouping

Participants were randomized to receive quetiapine monotherapy (*n* = 10) or TAU (standard of care psychotropics, *n* = 10). All participants received ten 90-min sessions of PE therapy with a staff psychologist as part of comprehensive rehabilitation services at the PRC.

### Participants

We planned to recruit 20 veterans at the age of 18–65 years, without sex restriction (10 per treatment group). Other inclusion criteria includes:

1. PTSD diagnosis, based on Clinician Administered PTSD Scale for DSM-5 **(**CAPS-5) of 25 or greater at screening.
2. History of mTBI as per VA/DoD guidelines; specifically, eligible participants must have at least one of the criteria among (a) loss of consciousness up to 30-min, (b) alteration of consciousness for up to 24 h, and (c) posttraumatic amnesia up to 1 day.
3. PCS diagnosis as described by ICD-10, i.e. having at least three of the following: sleep disturbance, irritability, headache, problems in attention/memory, fatigue, dizziness, and intolerance of stress, emotion, or alcohol use criteria.
4. Ability to read and write English.

Exclusion criteria includes:

1. Pregnant or lactating women and those of child-bearing potential not using a reliable method of contraception;
2. Co-morbid schizophrenia, bipolar disorder or schizoaffective disorder (as determined with DSM-5);
3. A history indicative of major neurocognitive disorder (dementia) or intellectual disability as determined by the investigator;
4. Currently taking any typical or atypical antipsychotic medication;
5. Known intolerance to quetiapine;
6. A history of clinically unstable heart, lung, liver, renal or endocrinological condition, diabetes mellitus, and/or seizure disorder;
7. Substance use disorder severe enough to require detoxification or inpatient hospitalization in the past month;
8. Current, ongoing serious suicidal or homicidal risk as assessed by the investigator;
9. Current or known history of cardiac arrhythmia or QTc interval ≥ 450 milliseconds;
10. Chronic daily benzodiazepine use;
11. Current engagement in other psychotherapeutic treatment.

The trial is conducted within the San Antonio Polytrauma Rehabilitation Center (PRC), which is housed within the South Texas Veterans Health Care System in San Antonio. Participants are recruited by referrals from rehabilitation and mental health staff in the PRC. The center provides a continuum of care for patients with TBI and other poly-traumatic injuries, including the post-acute phase of specialized rehabilitation services for those who have experienced mTBI. The TBI clinic within the rehabilitation center has implemented a collaborative care model that includes a neuropsychiatrist to provide medication and clinical psychologists to provide trauma-focused therapy to the patients with PTSD, which is the most common psychiatric diagnosis (90%) within the clinic. The VHA Support Service Center (VSSC) diagnosis cube shows that PRC San Antonio sees more than 300 veterans with comorbid mTBI and PTSD per year.

### Procedure

**Fig. 1:**
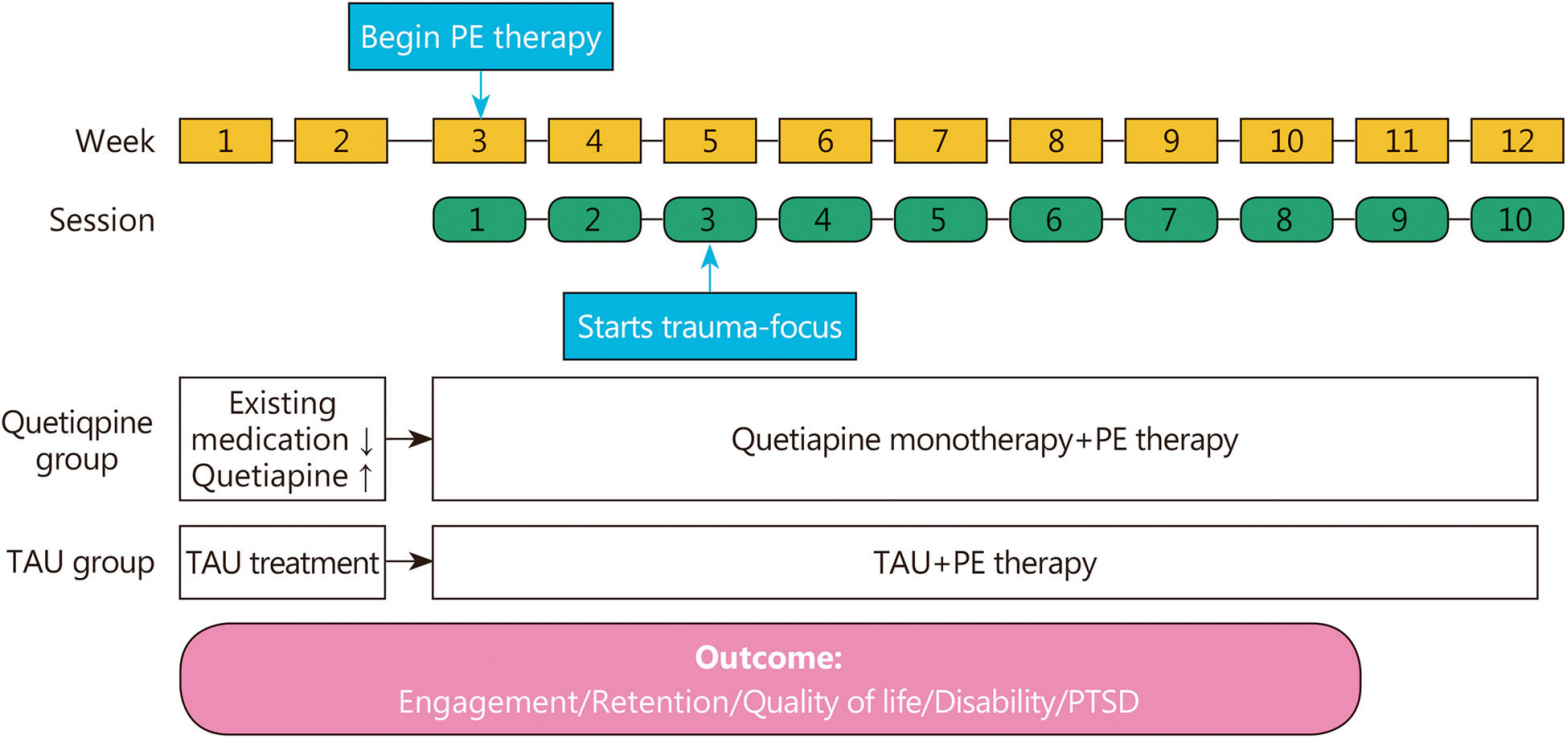
Study design: RCTs for Quetiapine vs. TAU treatment. TAU. Treatment as usual

Subjects were randomized to continue currently used medications (TAU group) or initiate quetiapine monotherapy. In the quetiapine group, currently used benzodiazepines, antidepressants, and other psychotropics were gradually reduced and eventually discontinued. Quetiapine dosage began at 25 mg at bedtime and was titrated up to 200 mg daily based upon tolerability and clinical response to maximize engagement in rehabilitation treatment. PE therapy started 2 weeks after randomization and lasted for a total of 10 weekly sessions. Quetiapine and TAU were maintained throughout the 12-week study. Doses were adjusted as clinically indicated (Fig. 1). Participants were allowed to continue other medications for general medical conditions, as well as anti-epileptic medications (e.g., divalproex, levetiracetam and carbamazepine) for seizure disorder or post traumatic headaches if the treatments lasted for at least 4 weeks prior to screening. A full list of study measures and assessment procedures is outlined in Table 1.

**Table 1:** Schedule of visits and assessments

Assessments	V0	V1	V2	V3	V4	V5	V6	V7	V8	V9	V10	V11
	Scr	WK1	WK3	WK4	WK5	WK6	WK7	WK8	WK9	WK10	WK11	WK12
Informed consent	X											
Med history & physical exam	X											
CAPS-5	X											X
PCL-5	X	X	X	X	X	X	X	X	X	X	X	X
PSQI	X	X	X	X	X	X	X	X	X	X	X	X
PHQ-9	X	X	X	X	X	X	X	X	X	X	X	X
QOLS	X											X
WHODAS 2.0	X											X
PTGI	X											X
Medication diary	X	X	X	X	X	X	X	X	X	X	X	X
Medication dosing		X	X	X	X	X	X	X	X	X	X	X
Medication adjustment		X	X		X				X			X
Adverse effects			X	X	X	X	X	X	X	X	X	X
Psychotherapy			S1	S2	S3	S4	S5	S6	S7	S8	S9	S10
Self-report of engagement			X	X	X	X	X	X	X	X	X	X
Vital signs	X	X	X	X	X	X	X	X	X	X	X	X
Labs	X											X
ECG	X											X

Shown are procedures completed at visits (V#) / weeks (WK) of study. Screening (Scr) occurs at V0 and medication reduction or initiation occurs from Weeks 1–3

*Abbreviations: CAPS-5* Clinician-Administered PTSD Scale for DSM-5, *PCL-5* PTSD Checklist for DSM-5, *PSQI* Pittsburgh Sleep Quality Index, *WHODAS 2.0* World Health Organization Disability Assessment Schedule 2.0, *QOLS* Quality of Life Scale, *PHQ-9* Patient Health Questionnaire, *PTGI* Post Traumatic Growth Inventory, *ECG* 12-lead electrocardiogram

PE therapy lasts for 10 weekly sessions, with trauma exposure starting at session #3 (Visit 4). A significant number of PTSD patients do not engage in the treatment sufficiently to begin session #3, and drop-out rates at that point or in subsequent trauma exposure sessions are high [71]. We have established two measures to promote engagement. First, PE treatment continued at least until session # 4 (i.e., after initial exposure session). Second, engagement was self-reported by participants using an 11-point emotional engagement scale (0: not engaged at all; 10: fully engaged) at every visit. A score of 7 or higher is desirable. Non-compliance with the PE treatment was also reported by the therapist.

The primary outcome was trial feasibility. We collected information on number of potentially eligible veterans approached, the number screened, and the number randomized. This information will allow us to determine patient acceptability, and the logistic feasibility of recruiting participants into a full-scale, open-label randomized trial.

PTSD Checklist for DSM-5 (PCL-5) is a 20-item self-report measure to screen individuals for PTSD, make a provisional PTSD diagnosis, and monitor symptom change during and after treatment [72]. The PCL-5 rating scale is 0–4 for each symptom: 0 = Not at all, 1 = A little bit, 2 = Moderately, 3 = Quite a bit, and 4 = Extremely. Total score of 34 is considered an optimal cutoff level for the diagnosis of PTSD [73]. Evidence has suggested that 10 point change in PCL score represents reliable and clinically significant change [72].

The 16-item QOLS self-report questionnaire was completed by the participants at screening and end of study (Visit 11) [74]. It is a measure of conceptual domains of quality of life: material and physical well-being, relationships with other people, social, community and civic activities, personal development and fulfillment, independence, and recreation. For each item, the score ranges from 1 to 7: 7 = Delighted, 6 = Pleased, 5 = Mostly Satisfied, 4 = Mixed, 3 = Mostly Dissatisfied, 2 = Unhappy, and 1 = Terrible. Total score ranges from 16 to112 [74].

The 12-item WHODAS 2.0 self-report was completed by the participants at screening and end of study (Visit 11) as a measure of functional disability [75]. It assesses 6 domains of functioning: cognition, mobility, self-care, getting along, life activities, and participation. For each domain, the score ranges from 0 to 4: 0 = No Difficulty, 1 = Mild Difficulty, 2 = Moderate Difficulty, 3 = Severe Difficulty, and 4 = Extreme Difficulty or Cannot Do. Total score ranges from 0 to 36.

Secondary outcomes included the Clinician-Administered PTSD Scale for DSM-5 (CAPS-5) [76]. We enrolled participants with CAPS-5 score of 25 and repeated assessment at end of treatment to determine clinically significant change. PTSD diagnosis was determined using the PTSD diagnosis algorithm recommended by the National Center of PTSD and requires at least moderate ratings (2 or more) on at least 1 B item (items #1–5), 1 C item (items #6–7), 2 D items (#8–14), and 2 E items (items #15–20) of the CAPS-5. Other secondary outcome measures included: the Pittsburgh Sleep Quality Index (PSQI), [39, 40, 77] the Patient Health Questionnaire (PHQ)-9 for depression severity, [78] the Post Traumatic Growth Inventory (PTGI) for cognition [79]. Adverse events (AEs) were documented (Table 1).

We assessed the impact of preconceptions about effectiveness of quetiapine on outcome measures. We collected this information from study participants, the study physician, and the study coordinator. Subjects are asked if they think quetiapine or TAU is preferable. If their response was yes, they were asked the reason for that belief. This assessment was completed before randomization, during, and after treatment, and used to assess possible bias by covarying the extent of those beliefs/attitudes/perceptions as a predictor of outcome.

Prescreening was accomplished by review of patient’s medical record, speaking to the patient’s treating clinician, and interview by the study coordinator in person (if possible) or by telephone to eliminate any obviously ineligible participants and determine the likelihood of eligibility. During chart review, lab data from clinical care informed study coordinator regarding an individual’s likely eligibility.

After prescreening, potentially eligible individuals were scheduled for written informed consent and a formal screening visit. After completion of the informed consent process, the study coordinator checked vital signs, and asked participants to complete the scales. The study physician performed a medical and psychiatric review with emphasis on traumatic experiences during childhood or military career, physical examination, and a diagnostic assessment using the CAPS-5. Laboratory tests included a standard hematologic and chemistry panel, liver and thyroid function tests, a urine drug screen and pregnancy test for females of child bearing potential only, and a 12-lead ECG. Participants who passed the eligibility screening were scheduled for randomization as soon as possible after screening. At the Randomization Visit, the study coordinator asked participants to complete self-report rating scales and then checked vital signs. The study physician then confirmed continued interest and eligibility for study participation before providing the research study medication dosing instructions.

Randomization was conducted in permuted blocks of 4. Randomization was stratified based upon whether PCL-5 scores were above or below an institutionally measured median score of 55 to assure that quetiapine/TAU groups were balanced on PTSD severity.

### Materials

Study medication supply were prepared and dispensed by the VA outpatient pharmacy. TAU group continued their prescribed medication and received dosages adjusted by the study physician as clinically indicated for irritability, anxiety, and sleep disturbances. Quetiapine was initiated at the dose 25 mg at bedtime and then increased in 25–50 mg steps up to 200 mg daily as clinically indicated over a 1–2 week period.

The participants randomized to quetiapine group who were taking standard of care psychotropic medications had their medications tapered off over a 2-week period as per clinical guidelines. We expected medications such as SSRI’s, SNRI’s, atypical antidepressants such as bupropion, mirtazapine, trazodone, and/or prazosin as most commonly prescribed medications for the treatment of PTSD. Trazodone, mirtazapine, bupropion, and prazosin were replaced with quetiapine at the time of initiation of quetiapine to minimize disruption in sleep while SSRI’s and SNRI’s were tapered off with lowering the dosage every 3 days to avoid serotonin withdrawal syndrome.

After at least 14 days of dosing (at Visit 2, beginning of Week 3), participants began PE with a VA trained therapist for 10 weekly sessions. We anticipated the highest need for medication adjustment during weeks 3 to 6 (visits 5–8) of PE therapy when in vivo and imaginal exposure of traumatic events started. The psychiatrist made dose adjustments based upon perceived need and patient tolerability. Study medication was dispensed 2 weeks at a time during visits 4–8. After visit 8, participants were given 4-week supply until the end of study visit. Dose adjustments were made in 25–50 mg increments and we anticipated most of the participants received dosage between 100 to 200 mg.

Quetiapine may cause sedation, and thus could help to normalize circadian rhythm when given at bedtime. The medications in the antipsychotic class prescribed at high dose, for longer duration of treatment, in severely mentally ill individuals increase the risk for diabetes and heart diseases by causing metabolic dysregulation. Our study population is thoroughly screened for metabolic risk factors by medical history and physical examination, vital signs, ECG, and basic laboratory tests. Quetiapine was prescribed at relatively small doses (i.e., up to 200 mg) for a short duration (12 weeks). Quetiapine has also been shown to cause prolongation of QTc interval. This is an issue usually in the individuals with known cardiac conditions and history of arrhythmias. This was avoided by excluding veterans with prolonged QTc ≥ 450 milliseconds. Basic laboratory tests to assess metabolic parameters and ECG were repeated at the end of study (Visit 13) to verify whether or not adverse changes were observed in these parameters. Dopamine blockade properties can cause extra-pyramidal symptoms, but it is least common with quetiapine compared to other anti-psychotic medications. Quetiapine use has been associated with respiratory dysfunction in patients with obstructive sleep apnea, a comorbid condition in severe and untreated mTBI and PTSD patients. Study participants were screened for sleep apnea and compliance with continuous positive airway pressure (CPAP) use was assessed and encouraged at every visit. At risk non-compliant participants were discontinued from further participation in the study.

At STVHCS, PE therapy was delivered in ten 90-min sessions following the model established by Foa et al. [80]. Patients are expected to complete in vivo and imaginal exposure exercises, breathing exercises, and listen to the session recording as homework to benefit from the therapy. In vivo exposure to safe situations, activities, places, and objects that patient usually avoids because of trauma-related anxiety and distress is introduced in session 2. An in vivo exposure hierarchy is constructed of specific situations, activities, places, and objects which the patient avoids. Imaginal exposure is revisiting the trauma memory in imagery which begins in session 3. Throughout therapy sessions 3–10, therapy always involves exposure to the target trauma and patients are assigned homework to be completed before the next visit throughout the course of therapy. PE works by engaging participants in the confrontation of safe but physiologically activating situations in order to overcome their excessive fear and anxiety. In the initial stages of therapy, the participants may experience increases in panic and anxiety during the sessions when confronting traumatic memories, situations, or thoughts which are distressing and which patients typically avoid. This is why we hypothesize that psychosedation with quetiapine should help patients to engage PE.

A total of 11 visits completed over 12 weeks comprise the study period. Briefly, the study coordinator met the participants at each of their weekly therapy visits, check vital signs, and collect weekly assessment. A brief electronic medical record review was conducted for pertinent health related issues and therapist’s report on the last session.

The study coordinator asked participants about medication adherence and the participant’s study medication bottles were checked and pills counted as a measure of medication adherence. Participants who did not initiate and continue study medication for at least 2 weeks or first PE therapy session were terminated. The study coordinator collected and recorded side effects, emergence of withdrawal symptoms, worsening of PTSD symptoms, or suicidal ideations data from participants every week and the study physician were alerted to clinically manage side effects and implement remedial procedures as indicated.

Participants were provided with emergency contact information for the study staff. In the circumstances, such as hospitalization or serious side effect such as suicidal or homicidal ideations participants contacted the study staff. If the situation required, then participants were transferred to an emergency department or inpatient psychiatric unit. Participation in the trial was terminated and a study discontinuation visit was scheduled when feasible. Participants were thanked for study participation and the study physician coordinated indicated care with the mental health service.

### Data analysis

This pilot study was not designed for efficacy or mechanistic hypotheses testing [81, 82]. Our primary interest was to evaluate feasibility of the proposed recruitment, treatment, and assessment protocols. As a rough guide to future planning, conventional effect sizes were calculated with 95% confidence limits [83]. For dimensional scales (e.g., PCL-5, QOLS, WHODAS 2.0, CAPS-5), effect sizes are model-based estimates of pre-post treatment change divided by baseline standard deviations (e.g., bias-adjusted Hedges’ g). Cohen’s index (h) [84], odds ratios, and number needed to treat (NNT) will be calculated for proportions. Although the statistical power of this study is limited, we will perform statistical analyses appropriate for an adequately powered study to identify data analysis issues germane to future planning, e.g., data management and scoring, missing data, data distributions, outliers, nature of trends over time, covariance structures. Statistical analyses will be intent to treat. We will use descriptive statistics to describe the study sample. For dimensional measures related to clinical outcome (i.e., PCL-5, PSQI), comparison of means will be done with general or generalized linear mixed effects regression models with repeated measures, with fixed effects of treatment, time, and the treatment by time interaction (e.g., SAS MIXED, GLIMMIX).When measures are assessed only at two time points, the treatment by time interaction is a test of the difference in pre-post change (i.e., CAPS-5, QOLS, WHODAS 2.0, PTGI). When measures are obtained during treatment as well, those will be included in the analysis models. For dichotomous measures of treatment engagement, statistical analyses will be done using chi-square tests. To address attrition and missing data, participants had frequent follow-up to maintain contact with the study team. Participants and their clinicians choosing to discontinue treatment were asked their reasons for discontinuing treatment as soon as possible at the point of drop-out. The proposed likelihood-based analyses are valid when the assumption that data is missing at random (MAR) holds.

## Discussion

The current study planned to evaluate quetiapine augmentation of PE therapy in PTSD patients with a history of mTBI. A search of NIH serviced national clinical trials registry and database [85] identified no current trial of quetiapine in PTSD patients. Of the four previous trials focusing on PTSD, one was published in 2016 [44]. None of these trials studied the effects of quetiapine in combination with TFT.

We initially considered a placebo-controlled trial but abandoned this option because it would require discontinuation of all other psychiatric medications and require 50% of patients to receive only placebo resulting in a denial of psychotropic standards of care. We then considered a quetiapine vs. placebo add-on to existing standard of care medication but decided against that strategy for two reasons. First, the sedative effects of quetiapine could interact with other sedating standard of care medications. Second, standard of care medications could potentially detract from the benefits of quetiapine monotherapy. We also considered blinding standard of care medicines by compounding these drugs into gelatin capsules prepared by the VA research pharmacist and medication dispensed in equal number of capsules to both quetiapine and TAU group. The effort-level required by this approach raised several issues related to both logistic feasibility as well as to the integrity of the blinding. We then considered the provision of adding back certain allowable standard of care medications open label to rescue breakthrough anxiety, sleep, and depression. However, this leaves open the possibility that added rescue medicines will interact with CPT outcomes and confuse interpretability of data. We have instead embraced a randomized, open-label design comparing quetiapine monotherapy with TAU polypharmacy practices that exclude use of quetiapine. We also considered quetiapine treatment for augmentation of cognitive processing therapy (CPT), but chose PE which has the strongest evidence-bases for treatment of PTSD [86] and has the highest likelihood of activation of arousal or negative emotional reactions [87]. At the PRC San Antonio among the standard of care treatments of PTSD, veterans referred for PE therapy have highest initial rejection and drop-out rate. More than 50% of veterans referred for PE therapy at the PRC rejected it and less than 30% completed the required number of PE sessions in year 2018, once therapy was initiated.

We expect that the success of this ongoing study should provide us with the preliminary data necessary to achieve funding for a full-scale randomized trial. This project is innovative in that it challenges the presumption that psychosedative medication effects may be counter-therapeutic.

## Data Availability

Final data sets underlying all publications resulting from this pilot study will be shared pursuant to a Data Use Agreement (DUA) appropriately limiting use of the dataset and prohibiting the recipient from identifying or re-identifying (or taking steps to identify or re-identify) any individual whose data are included in the dataset. We will make sufficient data and descriptors available to confirm conclusions in the publication, run duplicate statistical analysis, and perform additional analyses.

## Abbreviations

CAPS-5: Clinician Administered PTSD Scale for DSM-5
DSM-5: Diagnosis and Statistical Manual of Mental Disorder, Fifth Edition
ECG: Electrocardiogram
GABA: Gamma-aminobutyric Acid
ICD-10: International Classification of Diseases, 10th Edition
IOM: Institute of Medicine
IRB: Institutional Review Board
ITT: Intent to Treat
mTBI: Mild Traumatic Brain Injury
MAR: Missing at Random
PCL-5: PTSD checklist for DSM-5
PCS: Post-concussion Syndrome
PE: Prolonged Exposure
PHQ-9: Patient Health Questionnaire-9
PRC: Polytrauma Rehabilitation Center
PSQI: Pittsburgh Sleep Quality Index
PTGI: Post Traumatic Growth Inventory
PTSD: Posttraumatic Stress Disorder
REM: Rapid Eye Movement
SC MIRECC: South Central Mental Illness Research, Education, and Clinical Center
SSRI: Selective Serotonin Reuptake Inhibitor
STVHCS: South Texas Veterans Health Care System
TAU: Treatment as Usual
TBI: Traumatic Brain Injury
TFT: Trauma-focused Therapy
QOLS: Quality of Life Scale
QTc: Corrected QT interval
UTHSCSA: University of Texas Health Science Center at San Antonio
VSSC: VHA Support Service Center
WHODAS 2.0: World Health Organization Disability Assessment Scale
